# Supplementary material for: DMRforPairs: identifying Differentially Methylated Regions between unique samples using array based methylation profiles
Source: BMC Bioinformatics. 2014 May 15;15:141. doi: 10.1186/1471-2105-15-141 (PMC4046028; doi:10.1186/1471-2105-15-141)
Supplement: Additional file 2 — DMRforPairs output for the comparison of A431-MCF7 and NA17018-NA17105. Please start from the HTML files in each folder. Available via the BMC Bioinformatics website. [file 1471-2105-15-141-S2.zip › 1394847754114233_MOESM2_ESM/A431_MCF7/figures/9811.pdf]

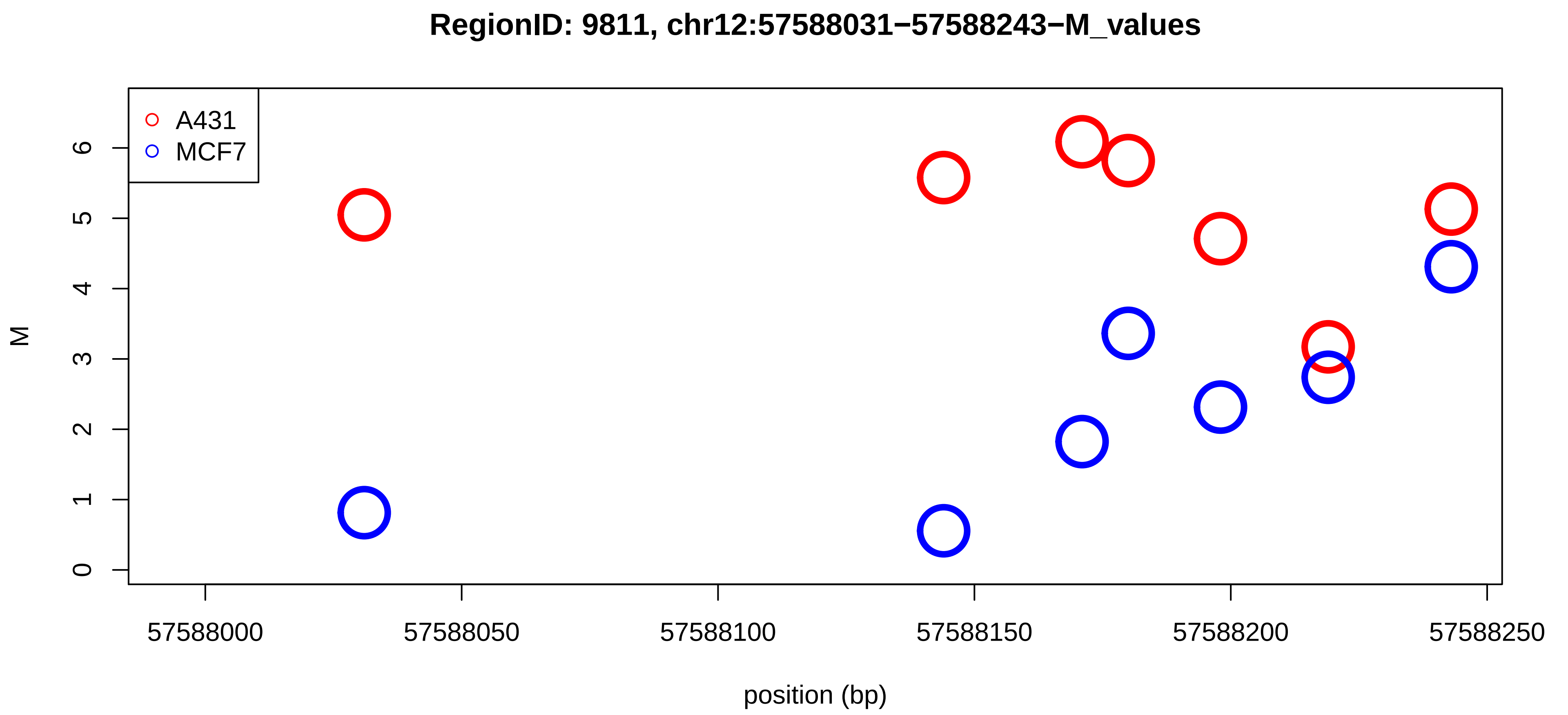

RegionID: 9811, chr12:57588031−57588243−M_values
A431
MCF7
M
0
1
2
3
4
5
6
57588000
57588050
57588100
57588150
57588200
57588250
position (bp)

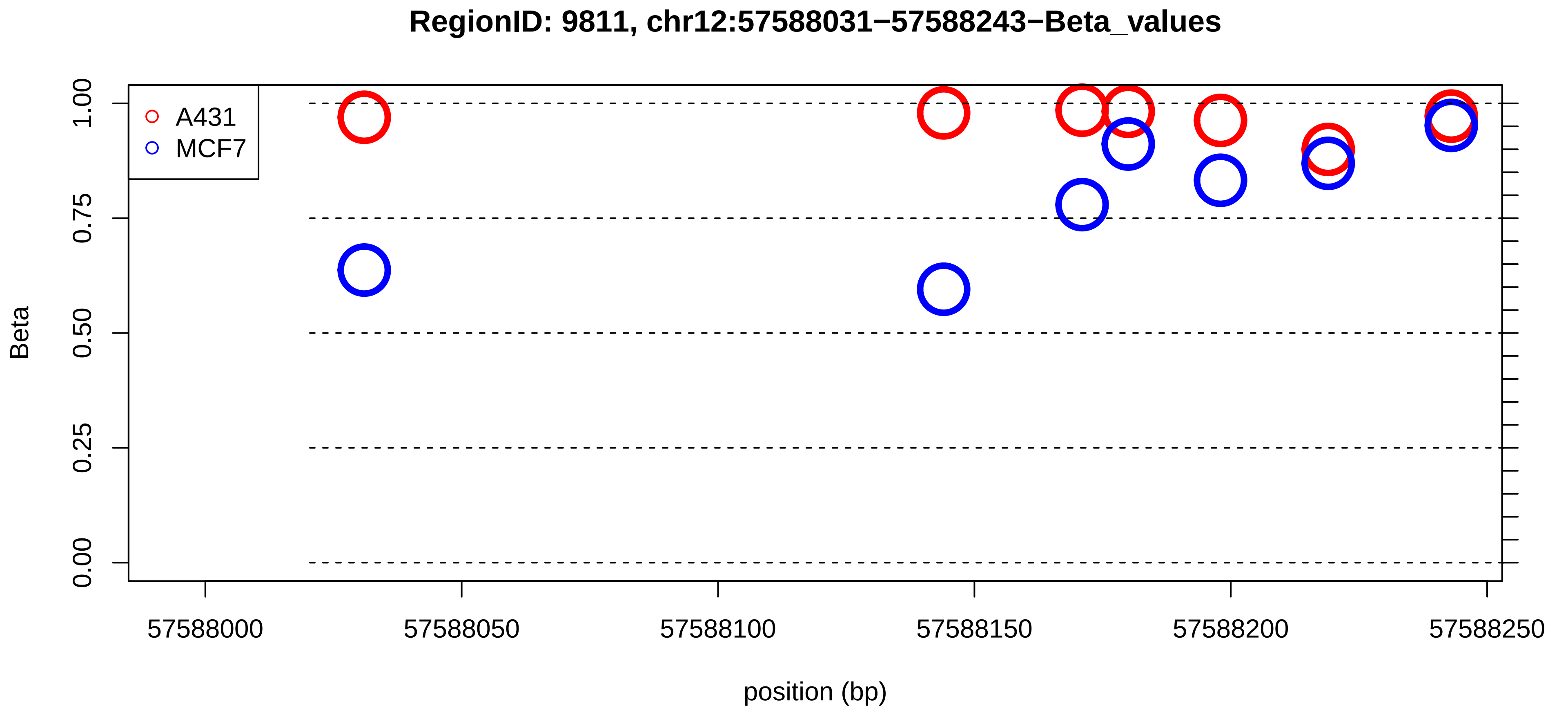

RegionID: 9811, chr12:57588031−57588243−Beta_values
A431
MCF7
Beta
0.00
0.25
0.50
0.75
1.00
57588000
57588050
57588100
57588150
57588200
57588250
position (bp)

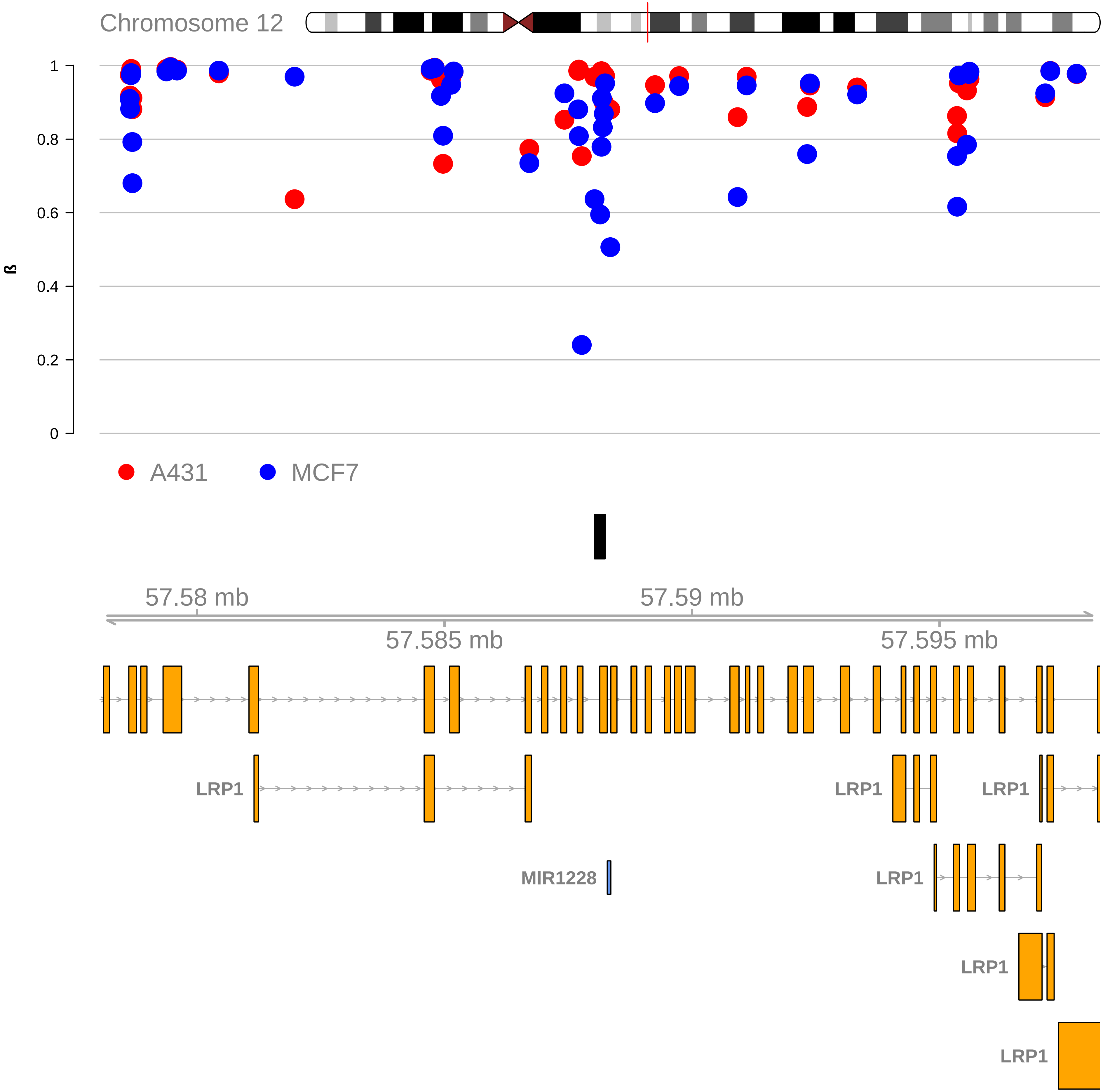

Chromosome 12
1
0.8
0.6
0.4
0.2
0
ß
A431
MCF7
57.58 mb
57.59 mb
57.585 mb
57.595 mb
LRP1
LRP1
LRP1
MIR1228
LRP1
LRP1
LRP1
